# Control of Neuroinflammation through Radiation-Induced Microglial Changes

**DOI:** 10.3390/cells10092381

**Published:** 2021-09-10

**Authors:** Alexandra Boyd, Sarah Byrne, Ryan J. Middleton, Richard B. Banati, Guo-Jun Liu

**Affiliations:** 1 Australian Nuclear Science and Technology Organisation, Sydney, NSW 2234, Australia; boyda@ansto.gov.au (A.B.); sarahbyrne916@gmail.com (S.B.); rym@ansto.gov.au (R.J.M.); rib@ansto.gov.au (R.B.B.); 2 Discipline of Medical Imaging & Radiation Sciences, Faculty of Medicine and Health, Brain and Mind Centre, University of Sydney, Sydney, NSW 2050, Australia

**Keywords:** neuroinflammation, microglia, TSPO, mitochondria, cytokines, antioxidants

## Abstract

Microglia, the innate immune cells of the central nervous system, play a pivotal role in the modulation of neuroinflammation. Neuroinflammation has been implicated in many diseases of the CNS, including Alzheimer’s disease and Parkinson’s disease. It is well documented that microglial activation, initiated by a variety of stressors, can trigger a potentially destructive neuroinflammatory response via the release of pro-inflammatory molecules, and reactive oxygen and nitrogen species. However, the potential anti-inflammatory and neuroprotective effects that microglia are also thought to exhibit have been under-investigated. The application of ionising radiation at different doses and dose schedules may reveal novel methods for the control of microglial response to stressors, potentially highlighting avenues for treatment of neuroinflammation associated CNS disorders, such as Alzheimer’s disease and Parkinson’s disease. There remains a need to characterise the response of microglia to radiation, particularly low dose ionising radiation.

## 1. Introduction

Ionising radiation (IR) as a diagnostic tool—such as X-ray, or positron emission tomography (PET)—and therapeutic technique has been widely used for decades in the pursuit of better health outcomes for patients [1]. Typically, these methods use lower doses of ionising radiation, and are prescribed when the potential benefits to receiving the procedure outweigh the risks associated with IR [2]. However, fundamental to this practice is the acceptance of the linear-no-threshold (LNT) model; the understanding that ionising radiation initiates detrimental effects to human health in a manner proportional to dosage [2,3]. This model, developed in the 1950s, arose from the extrapolation of the linear dose-response trend at higher doses and applying it to lower doses, where negative effects have been presumed, but not observed [3,4]. The LNT model has been employed by regulatory bodies and accepted by both scientific and medical communities in the absence of an alternate comprehensively proven model [5].

The acceptance of the LNT model may limit the potential of IR as a therapeutic tool except in instances where the benefits heavily outweigh the perceived risk, for example, radiotherapy. Although high doses of ionising radiation (HDIR) have been shown to have adverse health effects, such as carcinogenesis [6,7], the presumption that low dose ionising radiation (LDIR) would also have negative effects simply to a lesser degree is unfounded [8,9]. In fact, hormetic effects have been demonstrated in numerous aspects of human health; for example, sunlight is essential in vitamin D synthesis. However, high doses or prolonged exposures can result in sunburns and the development of skin cancers [10]. Additionally, data from both large-scale nuclear accidents [11,12], and the atomic bombings of Japan [13], do not fully support the LNT model, and there is a growing body of literature suggesting that exposure to LDIR may enhance putative neuroprotective adaptive cellular pathways, such as increased antioxidant levels and reduced reactive oxygen species, which may reduce inflammation within the CNS [14,15]. The increasing availability and utility of IR, and the growing body of evidence suggesting the invalidity of the LNT model, necessitates the reinvestigation of the current conceptions around the safety and dose limitations of IR.

Microglia, the resident immune cells of the central nervous system (CNS), respond to external stressors such as pathogenic invasion and injury by inducing inflammation [16,17,18]. During microglial activation, microglia are polarised from the M2 anti-inflammatory state to the M1 pro-inflammatory state [16,19,20]. Alterations in the functional states of microglia in response to stressors are characterised by morphological changes and functional plasticity [19,20]. The immune response that ensues is characterised by increased levels of pro-inflammatory cytokines and reactive oxygen species (ROS) which promote the degradation of damaged tissues and pathogenic invaders [21,22]. The M1 functional state is associated with pro-inflammatory cytokines such as IL-1β, IL-6, and TNF-α, whereas the M2 functional state is associated with anti-inflammatory cytokines such as IL-4 and IL-10 [23]. However, it has been shown that following microglial activation, both pro- and anti-inflammatory genes are upregulated [18].

Notably, the expression of translocator protein (TSPO) is upregulated within the mitochondria of activated microglia, and hence is often used as a biomarker of neuroinflammation [24]. This inflammatory effect though to be beneficial to the body, as it is a protective mechanism against disease. However, neuroinflammation has also been implicated in many CNS diseases, such as Alzheimer’s disease [25,26,27,28], depression [29], and Parkinson’s disease [30,31], indicating inappropriate chronic microglial activation. As microglia appear to play a critical role in the onset and maintenance of neuroinflammation, the physiology behind microglial activation and immune modulation pathways are of interest as potential therapeutic targets [32]. This review will examine the contrasting characteristics of activated microglia when exposed to differing degrees of stressors, with a focus on ionising radiation, to highlight the remaining uncertainties regarding microglial activation.

We acknowledge there is contention around both the term “neuroinflammation” and the diseases it applies to [33,34,35]. A large portion of the scientific community has embraced the term, applying it to any condition where microglial and astrocytic activation can be observed. This has led to the understanding that diseases such as Alzheimer’s disease, Parkinson’s disease, and depression are “neuroinflammatory” diseases [29,35,36]. However, gene expression data indicate that these diseases are distinct from other known inflammatory diseases [33,34,35]. Hence it best to restrictively use the term “neuroinflammation” as a shorthand to describe the presence of microglia whose morphology or RNA or protein expression profile is different from that ordinarily observed in health brain tissue. Since under the term “neuroinflammation” microglial state changes (or “microglial activation”) can be the consequence of a wide range of local or systemic immune system responses, “neuroinflammation” should not be used as predictors of specific physiological outcomes [33,34,35]. However, as the term neuroinflammation continues to be used by many authors, this review, too, will refer all instances of microglial activation as to neuroinflammation in its broader meaning but specify the context within which the term needs to be interpreted.

## 2. Functional States of Microglia Altered by Stressors

Microglial cells play an important role in inflammation, brain development, and the regulation of neuronal networks [37,38]. Historically referred to as the endogenous macrophages of the CNS, this description is not completely comprehensive as it reflects only one specific functionality of the cell and suggests that the mechanism of action for microglia and macrophages are inherently the same. However, the initiation and maintenance of an immune response is a major aspect of the function of microglial cells.

It is believed that microglia arise from primitive macrophages (myeloid progenitor cells) in the embryonic yolk sac of mammals, before infiltrating the brain where they differentiate and reside for life [39,40]. In the adult CNS, the microglial population does not arise from further myeloid progenitor cells, instead the resident microglia self-renew as needed and can rapidly proliferate in response to neural insults [41]. When a threat is detected, such as a pathogen or radiation injury, microglia undergo morphological transformations as they become “activated”. Traditionally, microglia were characterised as either active (M1) or resting (M2); however, it is now understood that a spectrum of microglial functional states exist [42,43]. Generally, active microglia adopt an amoeboid, less ramified morphology, allowing them to become more mobile and phagocytotic, whereas resting microglia have a smaller cell body and are highly ramified, allowing them to survey the microenvironment [43,44,45]. Morphological changes such as cell area, perimeter and ramification length are still frequently utilised in research as an indicator of the degree of neuroinflammation [46,47].

A third morphology is gaining further scientific attention. Dystrophic microglia tend to be small and de-ramified, with beaded or discontinuous processes [48,49]. The cause of dystrophic microglia remains unclear; however, it has been hypothesised that they are linked to ageing [16,48]. A recent study by Shahidehour et al. (2021) found that hypertrophic (activated) microglial numbers, and not dystrophic microglia numbers, were associated with ageing in the CA1 region of the hippocampus [49]. They found no difference in the percentage of hypertrophic microglia between neurodegenerative pathologies (AD, Lewy body dementia and limbic-predominant age-related TDP-43 encephalopathy) and age matched controls; however, in neurodegenerative pathology, 45% of microglia were dystrophic, compared to 9% in the control [49]. Ethanol exposure has also been shown to both reduce overall microglia numbers, whilst increasing the number dystrophic microglia in the hippocampus [50]. Interestingly, the researchers observed that the microglia following ethanol exposure appeared “activated but not to an M1-like, amoeboid state” highlighting the diversity of microglial functional states and how the M1/M2 classification system may need revision [50].

In addition to morphological changes during microglial activation, there are numerous alterations to bio-cellular pathways which promote an inflammatory immune response. These pathways have primarily been established by exposing microglial cells to lipopolysaccharide (LPS), an endotoxin derived from *Escherichia coli*. LPS, and other stressors, which act on toll-like receptor 4 (TLR4) of microglial cells, initiating an inflammatory response [51,52]. This triggers microglial cells to become phagocytotic; engulfing and degrading foreign materials [53]. It has recently been shown that inhibition of TLR4 in an Alzheimer’s cell model promotes an M2 phenotype and improves neurological function [54]. The triggering receptor expressed on myeloid cells 2 (TREM2) has also been implicated in the modulation of microglial phagocytosis, with some studies suggesting that TREM2 function, and hence phagocytotic function, may be impaired in instances of neurodegenerative diseases, particularly Alzheimer’s disease [55,56,57].

TLR4 signalling will lead to the downstream phosphorylation of Nuclear Factor Kappa B (NF-κB) inhibitory protein, promoting the expression of pro-inflammatory genes and therefore the expression of pro-inflammatory proteins [58,59,60,61,62]. Among the more commonly known ones are cytokines IL-1β [63], IL-6 [64], and TFNα [65] which play a role in triggering cell cycle arrest and apoptosis [66], the initiation of neurotoxicity [67], and inflammatory signalling [62,68]. There is also emerging evidence that some pro-inflammatory cytokines may enhance the dopaminergic differentiation of neural stem cells, i.e., they may possess neurogenic properties [69]. The transcription of enzymatic genes, such as *iNOS*, and apoptotic genes, such as *Fas-ligand*, results in the translation of proteins which play an active role in inflammation and cellular death [70,71,72]. In mice models of neurodegeneration, it has been observed that the downregulation of homeostatic microglial genes correlates with neuronal loss, whereas the upregulation of disease associated microglial genes did not [73]. The one exception to this was the *APOE* gene, which directly correlated with neuronal loss [73]. In fact, *APOE4* is a known genetic risk factor for Alzheimer’s disease [74]. The study also found that early AD pathology in the human brain was only associated with a loss of homeostatic genes, but not the gain of any disease related genes. This suggests interspecies variability in microglial gene expression during pathological states, meaning the results of murine studies may not translate into the clinic [73].

Increased reactive oxygen and nitrogen species (RONS) concentration are also a hallmark of neuroinflammation [75]. In microglia, the majority of RONS are reactive oxygen species generated via NADPH oxidase; however, they can also originate from other intra- and extracellular sources [76,77]. Interestingly, some studies show that LPS- and a-Synuclein-induced neuroinflammatory responses are attenuated in NOX2 (an isoform of NADPH oxidase) knockout mice, indicating that NOX2 may play a role in microglial activation [78]. NADPH oxidase has also been implicated in cognitive dysfunction in experimental autoimmune encephalomyelitis, being demonstrated to prevent long term potentiation [79]. The iNOS enzyme in microglia and macrophages is responsible for the production of nitric oxide (NO), a precursor to reactive nitrogen species (RNS) which, in conjunction with reactive oxygen species, result in oxidative damage to lipids and proteins [80]. NO can also prevent cell division, often leading to cell death, by inhibiting an enzyme required for DNA synthesis, or by directly causing double stranded DNA breaks [81,82,83,84].

On the other hand, microglia display an array of neuroprotective effects. Immune surveillance is constantly occurring under homeostatic conditions, during which microglial processes and filopodia randomly survey the external environment searching for cues which may trigger an immune response [85,86]. Microglia also have a role in synaptic pruning and promoting neurogenesis [87,88,89,90,91]. Synaptic pruning allows for the removal of weaker synaptic connections, which promotes the development of stronger pathways, allowing for clear and direct signal transduction [92]. The importance of microglia in synaptic pruning is demonstrated through the knockout of Cx3cr1 receptors, a critical receptor for microglial migration. Cx3cr1 knockout in mice results in immature brain circuitry, which possess the electrophysiological hallmarks of undeveloped synaptic function [89]. A transcriptomic analysis also demonstrated that the microglial phagocytosis of human apoptotic cells initiated the expression of neurogenic-related genes, strongly suggesting that microglia modulate the process [93]. Recently, one study found that the supernatant of M2 microglial cells (containing molecule 15-deoxy-Δ12,14-prostaglandin J2) promoted neurogenesis and oligodendrogenesis [94], whereas another demonstrated the direct contact between a microglial cell and neuronal dendrites promoted synaptic formation [95]. It has also been shown that microglia are necessary to learning-dependent synaptic formation, and this plasticity is regulated via brain derived neurotrophic factor [96]. It is therefore clear that microglial have a role in neurogenesis.

## 3. Impact of Ionising Radiation on Healthy Brains by Altering Microglial Function States

Phenotypic changes to microglia can be achieved by the application of ionising radiation, the effects of which are widely believed to be dependent on the dosage and dose schedule. Knowledge of the effects of ionising radiation, primarily established through large scale nuclear events, indicate that HDIR is detrimental to human health [97,98]. The linear no threshold model arose from extrapolating this knowledge and applying to it LDIR [99]. The LNT model is still widely used by many radiation protection organisations today; however, there is an increasing need to re-evaluate this on the basis of recent evidence highlighting the potentially positive effects of LDIR. Currently, there is no consensus on what dose constitutes LDIR or (HDIR). Often, a low dose is defined as below 100 mSv; however, this does not take into account dose rate, cumulative dose or potential interspecies variability. As such, a variety of doses and their effects have been included.

Aside from the widely accepted risk of carcinogenesis, HDIR has detrimental effects on the human CNS, having been linked to the onset of cognitive dysfunction [100,101,102,103], deficits of spatial-temporal learning, and reduced memory (2–54 Gy) [104,105,106,107,108]. Ionising radiation has also been shown to cause demyelination (see review [109]), and to disrupt neurogenesis (see reviews [110,111]). Additionally, a reduction in functional connectivity in the anterior cingulate cortex and right insular region has been observed following radiation therapy in nasopharyngeal carcinoma patients, where 68–70 Gy was administered over 30–33 fractions [112]. These deficits tend to peak around 4 months post radiation treatment [113], and can be irreversible. However, it is important to note that most human epidemiological studies arise from opportunity and, therefore, in most human studies the disease which is being treated may be a confounding factor. Similarly, a lot of long-term effects are unknown in these cases due to the typically shorter life span of the patients.

Cell and animal research continue to help bridge the gap in areas which human studies cannot explore. Various studies have shown a range of microglial responses to high dose ionising radiation; notably that irradiation using a high dosage will elicit a neuroinflammatory response [18,114,115,116,117,118]. A dose of 0.5 Gy has been shown to increase the number of microglia in the hippocampus, compared to control and low dose ionising radiation (0.063 Gy); however, the microglia were less ramified than before [118]. A similar increase in microglial density has been found in the cerebellum following a 6 Gy dose [119]. Osman et al. recently observed that a dose of 8 Gy on the juvenile murine brain induced transient microglial activation, as demonstrated through changes in microglial morphology and density [18]. Microglial activation was also associated with a transient increase in apoptotic cell levels, as well as a simultaneous increase in both pro- and anti-inflammatory genes. Notably, the effects of the ionising radiation tended to peak around 6 hrs, after which they began to decline [18].

**Figure 1:**
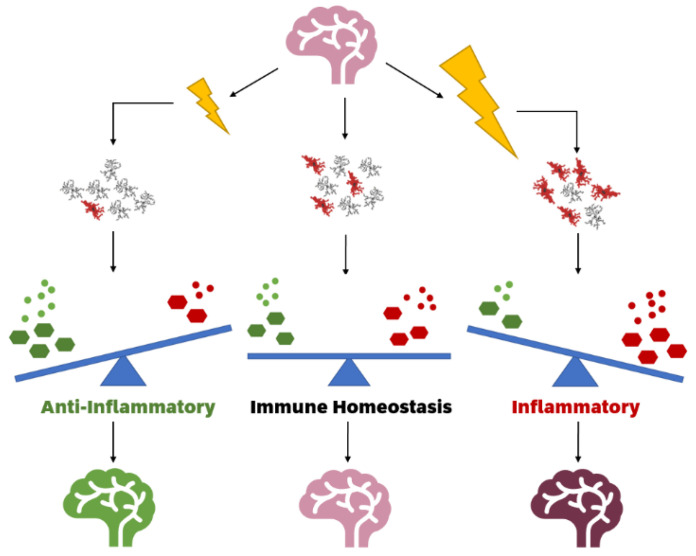
Ionising radiation modulates inflammatory response in a healthy brain by altering microglial functional states. Low dose ionising radiation (**left**) may reduce the number of activated microglia, increasing antioxidants and anti-inflammatory cytokines and thus having a neuroprotective effect when compared to a control brain (**middle**). High dose ionising radiation (**right**) increases the number of activated microglia, which increases oxidants and pro-inflammatory cytokines, creating a neuroinflammatory state.

Often linked with an increased microglial density is an increase in reactive oxygen and nitrogen species production, which leads to protein oxidation and lipid peroxidation [117,120,121,122,123,124]. Oxidative stress results in an increase in DNA damage such as double stranded breaks and concomitant decrease in DNA repair proteins [125] and diminished antioxidant enzyme activity [126]. However, one study contrasts this, showing that a dose of 200 mGy may reduce lipid peroxidation within the brain, with associated increases in catalase and antioxidant concentrations [127]. Pro-inflammatory cytokines such as IL-1β, TNF-α, and IL-6 have all been shown to be increased after irradiation and play a role in the inhibition of neurogenesis and further promotion of inflammation (refer to Figure 1) [106,117,119,128,129,130,131]. Other observed reactions that are of interest include changes in mitochondrial membrane potential and permeability [132], increased blood–brain barrier permeability [119,133], and the induction of pyroptosis [134,135]. Crucially, it has been shown that the transcriptome of microglia that have been exposed to high dose radiation is significantly similar to the M1 classical activation phenotype [136], indicating that HDIR activates microglia. Further evidencing the role of HDIR activated microglial in neuroinflammation are recent studies which demonstrate that acute pharmacological microglial depletion following radiation exposure alter the neuroinflammatory response and can prevent cognitive deficits [102,137].

It has previously been shown that ionising radiation alters the brain architecture in mice. In the hippocampus, ionising radiation has resulted in reductions in dendritic complexity and number, and has altered the concentration of synaptic proteins [138]. Recently, microglia have been associated with radiation-induced synaptic loss. Male mice exposed to 10 Gy IRexhibited a significant decrease in immature spinal density; however, the knockout of complement receptor 3 (CR3) was neuroprotective and prevented this loss [139]. Interestingly, female mice (knockout and wildtype) did not experience radiation induced decreases in spinal density, and also displayed a significantly higher number of intersections basally when compared to male mice, suggesting a sex-dependent effect [139]. This study also observed changes in microglial activation markers following the IR; however, not morphological changes, suggesting that morphological changes may not be a reliable indicator of microglial activation [139]. HDIR may also induce neuronal apoptosis by causing cell cycle arrest at the G2 and M checkpoints [117]. This study also indicated that the effects of HDIR may be delayed, with significant apoptosis and chemokine mRNA expression occurring 1-week post exposure [117].

Additionally, rodent models have allowed the investigation of the CNS effects of prenatal radiation exposure. The paucity of human studies have suggested there is potentially an increased risk of cognitive and health effects on a foetus; however, the Centers for Disease Control and Prevention (CDC, USA) has acknowledged that an acute radiation dose of <100 mGy has no observable non-cancer effects [140,141,142,143]. The majority of human studies which involve HDIR come from A-bomb survivors, and these indicate the potential for prenatal IR to cause mental retardation and microcephaly [144]. Animal studies have highlighted dose and pregnancy stage dependent effects of HDIR causing DNA damage, alterations to cell cycle checkpoints and apoptosis in the neocortex [145,146,147], and have identified behavioural/cognitive changes [145].

The effects of LDIR have been observed to be quite different and conflicting. There is a growing body of evidence which suggests that LDIR may be anti-neuroinflammatory (refer to Figure 1). This hypothesis is termed radiation hormesis. Human cells grown under reduced background radiation manage the stress of acute irradiation at high dose less efficiently than cells cultured under normal background radiation [148], supporting the theory that mild radiation exposure stimulates an adaptive response. Irradiated fruit flies and rodents also have shown enhanced immune systems and extended lifespans compared to non-irradiated controls [149,150,151,152]. One murine lifetime study found that an exposure of single 0.063 Gy radiation significantly reduces the risk of the development of many types of tumours, including pheochromocytomas, adenomas, insulinomas, and adenocarcinomas, compared to non-exposed controls [152]. LDIR is also thought to confer protection to cell functioning, molecular structures, synapses and key brain mechanisms such as neurogenesis, as well as inducing reparative functions (see review [153]). These theories have been supported by observed physiological responses LDIR. The suppression of ROS is one such example [154,155]. Studies of occupationally exposed workers found that chronic LDIR (0.1–8.4 mGy per month) was associated with an increased resistance to oxidative stress [156,157]. This effect has also been observed at higher acute doses, with acute 0.2 Gy IR increasing antioxidants in the blood and tissues of rats [127]. Increases in anti-inflammatory cytokines such as IL-4, IL-10, and reductions in inflammatory cytokines such as TFN-α have been observed at 50 mGy [158]; however, this effect has also been observed at higher doses of up to 1 Gy [25,159,160]. Of note, one study found that 100 mGy actually increased inflammatory cytokines [161], and another found that 100 mGy may have a negative effect on cognition, although noted further investigation is required [108]. Notably, 100 mGy appears to disrupt the BBB, which is a key aspect of neuroinflammation [74,162].

A recent study by Ung et al. (2020) specifically examined the effects of a singular radiation event on both murine glial cells and murine behaviour [118]. At the “high” dose of 0.5 Gy, there was an observable decrease in acoustic startle response, exploration, and rearing at 12 months post exposure. At 24 months post exposure, there was a significant increase in the number of microglial cells in the dentate gyrus, and a reduction in both astrocyte number and complex morphology. However, mice exposed to a singular dose of 0.063 Gy radiation had an increased acoustic startle response, increased exploratory behaviour, and increased rearing at 18 months post exposure, and had significant increases in microglial ramification at 24 months (compared to both the control and other irradiated groups of 0.125 and 0.5 Gy) despite no observable increase in microglial density. The astrocyte morphology of this group was also not significantly different to the control group. This study clearly demonstrates the potential for LDIR to be anti-neuroinflammatory in comparison to HDIR [118]. A complementary study by Hladik et al. (2020) observed the effects of a single radiation event on cAMP response element-binding protein (CREB) signalling; a transcription factor involved in memory formation, neuroplasticity and amyloid processing [163]. The CREB pathway, and other associated pathways, were found to be “activated” (as determined by alterations in hippocampal protein levels) by a dose of either 0.063 Gy or 0.125 Gy, whereas these pathways were “deactivated” by a dose of 0.5 Gy. Conditioning, learning, and long-term potentiation were found to be activated by 0.063 Gy or 0.125 Gy and were deactivated at 0.5 Gy. Additionally, a dose of 0.125 Gy was found to deactivate apoptosis. This data further supports the concept that LDIR may allow for neuroprotective cellular adaptive responses in comparison to the detrimental effects of higher doses [163], a premise which is supported by our recent study in press at *Frontiers in Cell and Developmental Biology*, which demonstrated that LDIR (10 mGy) may enhance neuroprotective pathways in the healthy brain [164].

However, radiation hormesis tends to be poorly supported by most human studies. An 11 million person cohort study found that the LDIR dose from a computed tomography (CT) scan (~40 mGy) during childhood and adolescent correlates with an increased incidence of cancers [165]. A recent meta-analysis examining a potential link between low dose ionising radiation exposure in adulthood and cancer similarly found that, after excluding studies with potential biases from the null, there was still a positive risk estimate reported by many studies [166]. Another systematic review found no positive effects of LDIR on neurodevelopment and cognition; however, it indicated that the evidence of adverse effects is “limited to inadequate” [167]. In a population of adults with congenital heart disease, a greater exposure to LDIR from cardiac procedures correlates with an increased cancer incidence [168]; however, the population of “adults without cancer” were significantly younger therefore had less comorbidities than the “adults with cancer population.” A review by Lumniczky, Szatmári, and Sáfrány (2017) found that LDIR could result in cognitive defects and other unfavourable outcomes in both human and animal populations, and resulted in the induction of different molecular and cellular mechanisms [169]. The authors called into question the safety of LDIR for diagnostic purposes [169]. Another review by Tang and Loganovsky (2018) concluded that LDIR (<100 mGy) or low dose rate ionising radiation (<6 mSv/hr) may or may not induce cancer, depending on a variety of factors including demographics, lifestyle, and diagnostic accuracy [170]. However, LDIR may increase incidences of vascular diseases, cognitive and mental health disorders, eye diseases, and other pathologies, whilst reducing cancer mortality and mutations, and increasing longevity [170]. Despite more studies beginning to investigate radiosensitivity [171], it remains unclear what effect LDIR truly has on different organs or tissues. One important effect which cannot be ignored is the potential effect of LDIR on cellular senescence. Carbon ion irradiation (1 mGy) can lead to premature senescence in human lung fibroblasts; however, this effect was not observed following 1 mGy gamma irradiation [172].

Given that ionising radiation can induce the polarisation of resting microglia into an activated state, there is the potential to explore different doses of ionising radiation as a tool to trigger the switching between activation states; in particular, from inflammatory to anti-inflammatory state by LDIR. We know that the functional state of microglia is dynamic, and that changing the environment can be a mechanism of manipulating them [173]. It has already been demonstrated that there is potential to convert microglia into an inflammatory, M1 phenotype then alter the environment to trigger a switch back to the M2 phenotype [174]. There is also evidence to suggest that the microglial activation state can be manipulated by repeated challenges with stimuli that influence future microglial behaviour upon subsequent stress [175]. Therefore, future studies should investigate whether challenging microglia with different doses and dose schedules of ionising radiation can enable the control of the microglial functional state, as it is a potential therapeutic tool for neuroinflammation-associated pathologies such as Alzheimer’s and Parkinson’s disease.

## 4. Impact of Low Dose Ionising Radiation on Neurodegenerative Diseases

**Figure 2:**
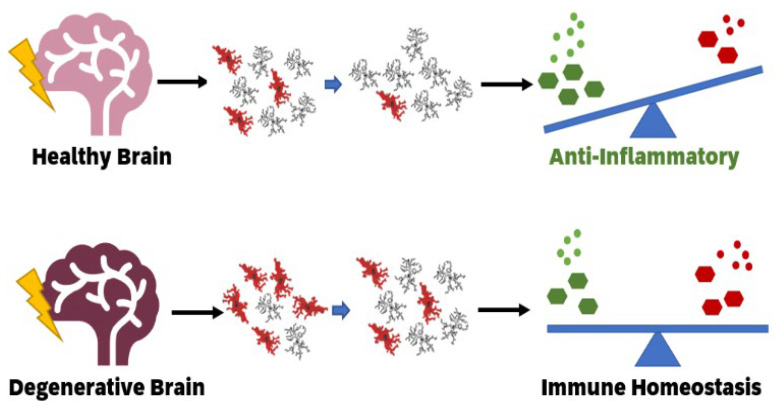
Low dose ionising radiation strengthens immunity in the healthy brain and reduces neurodegenerative disease by changing microglial functional states. A healthy brain exposed to low dose ionising radiation may experience anti-inflammatory effects, whereas in a degenerative brain, such as in Alzheimer’s disease, low dose ionising radiation may lessen the severity of neuroinflammation and promote a shift towards a more “normal” brain environment.

The positive effects of LDIR on models of Alzheimer’s disease have been seen in mice, particularly in transgenic AD female mice (refer to Figure 2) [176]. A dose of 100 mGy improved locomotor activity in Alzheimer’s-like transgenic (Tg) female mice, improved their grip strength and reduced Aβx-40 levels. The same dose in male Tg mice had few effects but did significantly reduce motor coordination. Interestingly, the higher dose of 500 mGy also improved locomotor activity in Tg females for open maze test and for Tg males in the Y-maze, improved motor coordination in Tg females and reduced Aβx-40 levels. Arguably, the most important takeaway from this study is that, without radiation, Tg female mice have higher Aβ levels than Tg male mice; however, radiation reduced these levels in the female Tg mice, which correlated with decreased microglial activation as determined by CD68 receptor staining [176]. Recently, rat models of AD were also shown to have improved memory performance in response to a higher dose of ionising radiation of 2 Gy/day for 5 days, without increasing neuroinflammation or amyloid load [177]. LDIR has also been shown to promote an M2 morphology in LPS treated mice microglial (BV2) cells [25].

Human case studies have used low dose ionising radiation as a treatment for neurodegenerative disorders (refer to Figure 2). The most well-known is a series of articles following a patient with end stage AD. After 2 CT scans (~40 mGy each) to detect anatomical changes, the patient displayed several behavioural changes noticed by her family and carers including signs of old memory return, improved motor function, and short three- to five-word sentence formation [178]. The patient then began to receive a CT scan approximately every 2 weeks. Interestingly, following the fifth CT scan the patient exhibited significant decrease in cognitive and motor abilities, potentially demonstrating the fine balance between both dosage and dose rate on whether radiation has advantageous or deleterious effects. The patient recovered from this setback, and continued to receive frequent, though more spaced out, CT scans [178,179,180]. Additionally, having observed these positive effects on his wife, the partner of the Alzheimer’s patient opted to receive CT scans to treat his Parkinson’s disease [179]. He received a CT scan every few months. The patient was able to reduce the dose of his medication, his tremors reduced, he was less constipated and his vision improved [180]. These promising results prompted a small pilot study where CT scans were used on four patients with AD [181]. Minor quantitative changes were observed; however, there were “remarkable improvements” in qualitative measures such as communication and behavioural changes. One of the four patients showed no improvement [181]. Together, this case and pilot study show the positive effects low dose radiation may confer on Alzheimer’s disease; however, until we can better control and understand LDIR as a treatment, it is not feasible to use therapeutically.

It is also important to consider the effects of radiation in multiple sclerosis (MS), where the immune system is already under the stress of an autoimmune condition. There is some evidence which may suggest that ionising exposure may represent a greater risk in MS than in healthy individuals. A clinical trial on total lymphoid irradiation (TLI) (19.8 Gy) for the treatment of MS found that irradiation reducing lymphocyte numbers to <900 mm^−3^ slowed the progression of the disease [182]. A follow-up found that any toxicity was “mild and transient” and suggested that the benefits of the treatment outweighed the disadvantages; however, it did indicate that menopause was induced in two patients and a staphylococcal pneumonia infection in another [183]. A further statement from the authors discussed the causes of five deaths among the cohort. All of the deceased were in a high lymphocyte, poor prognosis category, so it is plausible that the deaths may have occurred regardless; however, there is also the possibility that the risk for serious infections is increased after TLI [184]. The patient with the induced staphylococcal pneumonia infection died from aspiration, providing merit to the latter theory [184].

Additionally, a 2013 case report suggested that conventional doses of ionising radiation used to treat meningioma induced the onset of multiple sclerosis in a 43-year-old woman, suggesting a potential connection but not establishing a causal relationship [185]. Other case and cohort studies have indicated a similar trend [186,187,188,189]. One cohort study even found that X-ray exposure site was relevant, with chest X-rays, skull X-rays, and brain CT scans all aligning with a higher incidence of MS [190]. Conversely, there are studies that indicate that there is an inverse relationship between IR, specifically ultra-violet B (UVB) exposure, and the development of MS [191,192]. Gamma Knife Radiosurgery (85 Gy) has been used to treat trigeminal neuralgia in MS patients, with 82% of patients reporting a reporting a reduction in pain following one treatment [193]. However, the authors acknowledge this needs to be explored further, with increased radiation toxicity being observed by some studies [194]. One murine study observed that repeated 0.5 Gy doses (to a dose of 10 Gy) of gamma radiation reduced many autoimmune symptoms, including splenomegaly, lymphadenopathy, and proteinuria [195]. It therefore remains unclear the effects of ionising radiation in pre-existing immune conditions.

## 5. TSPO as a Biomarker for Changes in Microglia

To quantify microglial activation, appropriate biological markers are necessary. These biomarkers can take on a variety of forms, such as cell receptors or cytokines. The most commonly used marker of microglial activation is ionised calcium binding molecule 1 (Iba1), also known as allograft inflammatory factor 1. In the CNS, it is expressed solely by activated microglial cells and is responsible for membrane ruffling and phagocytosis [196]. Iba1 is highly conserved across species and can be easily detected through anti-Iba1 antibodies [197].

Pro-inflammatory and anti-inflammatory cytokines and chemokines can also be examined to elucidate the current activation state of microglia. Increased concentrations of pro-inflammatory molecules such as IL-6, IL-1β, IL-18, TNF-α, IFN-γ, CCL5, or GM-CSF all indicate a greater proportion of activated microglia, and hence likely a greater degree of neuroinflammation [198]. Opposingly, higher concentrations of IL-4, IL-10, TGFβ, or CCL22 all signify an anti-inflammatory environment [198]. However, cytokine and chemokine analyses are often used to complement Iba-1 data, rather than as standalone data, as they are not “neuroinflammation” specific and often require the use of homogenised tissues, leading to spatial information being lost [199]. Therefore, these molecules tend not to be the best biomarker of neuroinflammation.

Mitochondrial translocator protein 18 kDa (TSPO), like Iba-1, has been shown to be upregulated in microglia under conditions of stress and pathology [200,201,202,203] and serves as a biomarker of neuroinflammation [204,205], particularly for in vivo molecular imaging such as a PET scan [206]. TSPO has a low basal expression in the central nervous system, predominantly by endothelial cells [207]. Although its exact function remains unclear, TSPO knockout mice have both helped to disprove its role in cholesterol translocation and steroidogenesis [208,209,210,211], and highlight a role in microglial activation and mitochondrial function [212]. TSPO is an attractive target for studying the effects of ionising radiation as it has been implicated in ROS production and ROS-mediated oxidative damage [213,214], and the addition of TSPO ligands, such as PK11195 or Midazolam, have been shown to reduce pro-inflammatory gene expression, accompanied by a reduction in activated microglia [215,216,217]. Manipulating TSPO expression or function will allow the understanding of the link between mitochondrial function and neuroinflammation to strengthen, supporting the future development of therapeutics targeting these elements. The use of a TSPO knockout model to observe the behaviour of TSPO under varying conditions, and the potential impact on the CNS microenvironment, provides a unique tool to characterise microglial response in the presence and absence of TSPO to varying stressors and would be invaluable to future study [209]. Our recent study demonstrates that there are decreases in TSPO and Iba1 mRNA and protein levels in brain, and proinflammatory cytokine IL6 in blood plasma, following 10 mGy IR, and increases in TSPO protein expression at 2 Gy in the brains of healthy mice and in primary cultured microglia [164]. As the levels of neuroinflammation in the healthy brain are minimal, there is little inflammation to be reduced by low dose radiation. However, the clear trend towards downregulation of TSPO and Iba1 expression indicates that LDIR may reduce microglial activation, and hence neuroinflammation. Further investigations should be undertaken in models of neurodegenerative diseases, where elevated levels of neuroinflammation are observed.

One potential downside, elucidated by a recent study, is that neuronal activity increases TSPO levels within the brain, specifically in neurons, meaning it may be an unreliable measure of glial activation [218]. Additionally, the post-mortem brains of late stage AD and Dementia with Lewy Bodies have shown similar TSPO levels to age matched controls, and even showed a reduction in some areas such as the substantia nigra [219]. Here, a reduction in TSPO may not indicate a reduction in neuroinflammation, rather may reflect “dystrophy, senescence and death, or dysfunction of mitochondria in the microglia” [219]. Another recent study concluded that whilst TSPO effectively marks activated microglia, it is not a predictor of neuronal loss, and as such only marks neuroinflammation and not neurodegeneration [220]. Finally, there is evidence to suggest that TSPO ligands may bind to plasma proteins, and therefore are unavailable to bind to TSPO, affecting the accuracy of PET scan results regarding neuroinflammation [221].

## 6. Conclusions and Future Directions

As the innate immune cells of the central nervous system, microglia facilitate the initiation and maintenance of basic immunity and neuroinflammation. The activation of microglia through stressors such as radiation prompts the transcription of pro-inflammatory genes, leading to the release of molecules such as IL-6 and TFN-α and the adoption of an ameboid, phagocytotic morphology. However, there is a growing body of evidence that LDIR may not act as a stressor, rather that LDIR may confer neuroprotection. Whilst the molecular mechanisms behind this are largely unknown, many cellular and animal studies have found LDIR promotes longevity, neurogenesis, and cognition, while decreasing ROS production. There is paucity of human studies on the effects of LDIR on microglia, and hence neuroinflammation. As such, the current understanding of radiation hormesis is poor. As highlighted throughout the review, the dosage which divides the positive and negative effects of radiation is hard to determine, with many studies providing conflicting results. Furthermore, the duration (transient or indefinite) of any potential positive outcomes is undetermined, and the interactions between different doses and exposure frequencies are under-explored. It may be that case that repeated “low dose” treatments will accrue and transform a positive effect into a detrimental one, as seen in the case study where CT scans were used to treat a patient with AD [178]. Whilst the case studies of a woman with AD and a man with PD show promise in the potential treatment of neuroinflammatory conditions [179], the large cohort study of people who received a CT scan in childhood or adolescence indicated an increased cancer risk [165]. It remains unclear whether the effects of LDIR are beneficial to or adversely impact human health. Further animal and cell work is required to elucidate the mechanisms behind the observed microglial mediated neuroprotection, and research needs to be undertaken regarding dose-rate, age when exposed (adulthood vs adolescence) and whether the impact is not only beneficial in disease states but also to a healthy population.
